# Metagenome-mining indicates an association between bacteriocin presence and strain diversity in the infant gut

**DOI:** 10.1186/s12864-023-09388-0

**Published:** 2023-05-31

**Authors:** Ida Ormaasen, Knut Rudi, Dzung B. Diep, Lars Snipen

**Affiliations:** Faculty of Chemistry, Biotechnology and Food Sciences, Norwegian University of Life Sciences, Ås, Norway

**Keywords:** Bacteriocins, Genome mining, Infant gut microbiota

## Abstract

**Background:**

Our knowledge about the ecological role of bacterial antimicrobial peptides (bacteriocins) in the human gut is limited, particularly in relation to their role in the diversification of the gut microbiota during early life. The aim of this paper was therefore to address associations between bacteriocins and bacterial diversity in the human gut microbiota. To investigate this, we did an extensive screening of 2564 healthy human gut metagenomes for the presence of predicted bacteriocin-encoding genes, comparing bacteriocin gene presence to strain diversity and age.

**Results:**

We found that the abundance of bacteriocin genes was significantly higher in infant-like metagenomes (< 2 years) compared to adult-like metagenomes (2–107 years). By comparing infant-like metagenomes with and without a given bacteriocin, we found that bacteriocin presence was associated with increased strain diversities.

**Conclusions:**

Our findings indicate that bacteriocins may play a role in the strain diversification during the infant gut microbiota establishment.

**Supplementary Information:**

The online version contains supplementary material available at 10.1186/s12864-023-09388-0.

## Background

Gut colonization during infancy is a complex process, involving recruitment of strains and species until an adult-like gut microbiota is reached [1]. The diversity increases drastically the first years of life, and factors involved are the mode of delivery, antibiotic usage, microbial exposure, and diet [2–4]. However, the underlying ecological forces of the early bacterial recruitment and increasing diversity remain poorly understood [5]. The ecological forces shaping the gut microbiota have been considered to include the opposing selection pressures from the host and the bacteria themselves [6]. Orchestration by the host on the gut microbiota, such as host immunity control of the bacterial composition, is termed top-down selection [6]. Top-down selection has been suggested to be of the diversifying kind, ensuring stability and functional redundancy in the gut. On the other hand, bottom-up mechanisms are commonly considered to reduce diversity [6]. However, in cases of intransitive competition, bacterial-bacterial interactions may increase the diversity in a bacterial community [7]. Intransitive competition means that there is no dominant competitor among the species competing for the same resources. This allows coexistence between species in the same niche [8]. The most widely explored model for this is the rock-paper-scissors (RPS) based niche competition [9–12]. RPS dynamics is the simplest form of intransitive competition, modelling competition outcomes in a system with three competing components [13, 14]. Such a scenario, where there is no definite winner, can occur when mechanisms for competitiveness come at a high price [9]. An example of a costly, competitive strategy is the use of antimicrobial compounds [15]. Therefore, the diversification process of microbial communities can involve the production of antimicrobial compounds [10].

One of the most widely distributed antimicrobial compound produced by bacteria are bacteriocins [16]. Bacteriocins are ribosomally synthesized peptides or large proteins with antibacterial activity [17, 18]. These compounds are produced by bacteria and archaea to enhance their competitiveness for nutrients and ecological niches [19]. Bacteriocins can target and disrupt the cell membrane integrity, or inhibit transcription, translation, replication or synthesis of the cell wall [20]. They are divided into three classes (I-III) based on their structure, and each class includes several subclasses [18]. Since the bacteriocin databases BAGEL4 and BACTIBASE do not contain complete subclass information, we have not focused on subclasses in this article. Class I bacteriocins are small (< 10 kDa) post-translationally modified peptides, while class II bacteriocins are unmodified (< 10 kDa). Bacteriocins in class III are large (> 10 kDa) and heat-lable, often with enzymatic activity [20]. Bacteriocin genes of all three classes have been identified in bacteria from different sites of the human body, including the gut [21].

Genome mining projects have discovered a vast variety of gut bacteria with the potential to produce bacteriocins [21, 22]. Cultivating experiments of fecal samples from infant guts and mother’s milk have resulted in findings of bacteria with antimicrobial activity, demonstrating that the infant gut accommodates bacteriocin-producing bacteria [23–27]. The bacteriocin producers may contribute to the formation of the infant gut microbiota as it has been shown that addition of bacteriocins to a gut bacterial community modulates the bacterial composition [28, 29]. However, to our knowledge, a complete characterization of bacteriocin-producing bacteria in the infant gut microbiota, from a microbial ecological point of interest, has not been performed. Thus, there is a need for extensive exploration of bacteriocins and their ecological role in the infant gut.

**Fig. 1:**
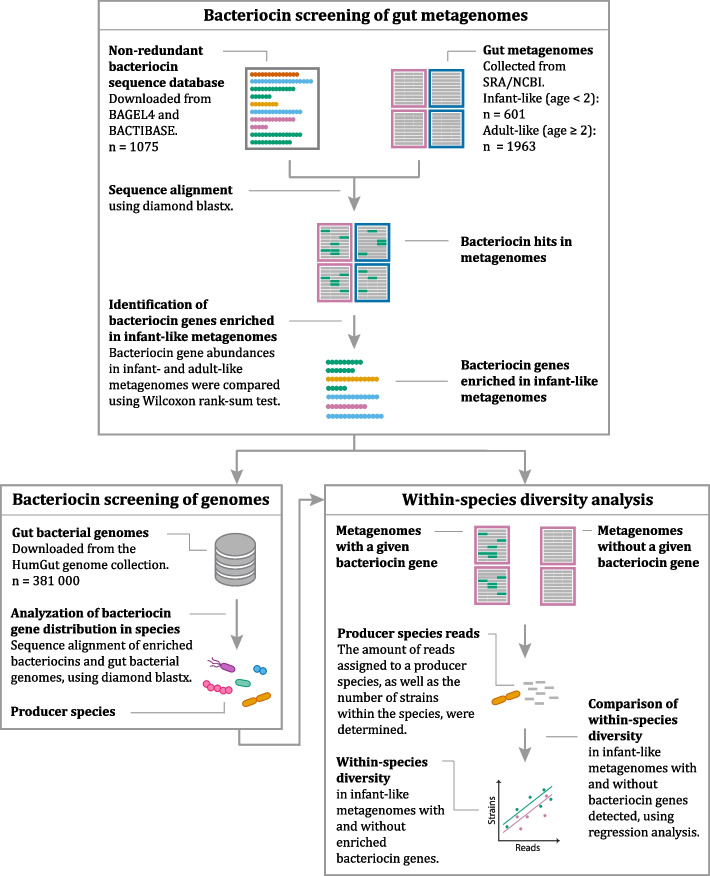
Searching strategy. Human gut metagenomes as well as the genomes in the HumGut collection were scanned for bacteriocins. A search for bacteriocins enriched in infant-like metagenomes was accompanied by the determination of bacteriocin-associated taxa. Further, the strain diversity within the bacteriocin-associated taxa was determined for the infant-like metagenomes and compared between metagenomes with and without the bacteriocins detected

The objective of this study was to test the idea of that bacteriocins can play a role in the gut microbiota establishment and diversification during the transition from an infant- to an adult-like microbiota. This was done by performing a comprehensive bacteriocin screening of gut metagenomes from healthy people to identify and characterize bacteriocins that are enriched in infants and investigate their potential role in association to composition and diversity of the infant gut microbiota. We collected all bacteriocin sequences from two public databases [30–32] and searched for their presence in publicly available gut metagenomes from healthy persons, as well as in the HumGut catalogue of human gut genomes [33]. An outline of the strategies used in this paper is shown in Fig. 1.

Our main findings were that infant-like metagenomes contain a higher abundance of bacteriocin genes compared to adult-like metagenomes, and that the strain diversity in the infant-like metagenomes was higher if bacteriocin genes were present compared to if they were absent. These results indicate that bacteriocins are of importance in the early gut microbiota formation, possibly by promotion of strain diversity.

## Results

### Overall distribution of bacteriocin genes in gut metagenomes

We executed a bacteriocin search (diamond blastx) in 2564 metagenomes from healthy persons consisting of more than 8 billion reads in total, with a collective size of around 10 terabases, using all the 1075 unique bacteriocin sequences from the bacteriocin databases BAGEL4 and BACTIBASE. Overall, 5.1 × 10^6^ bacteriocin-matching reads were identified (E-value below 10^–5^).

### Microbial diversity and distribution of bacteriocin genes in gut metagenomes

**Fig. 2:**
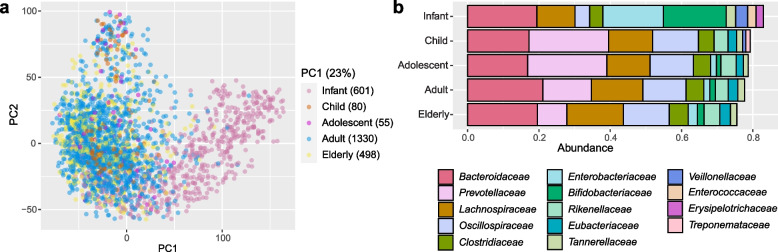
Bacterial diversity of the metagenomes. In panel (**a**), the beta diversity of the metagenomes in the infant group (pink), the child group (orange), the adolescent group (purple), the adult group (blue) and the elderly group (yellow) is illustrated in a Principal Component Analysis. Each dot corresponds to the species profile (read abundances for each species) for each metagenome, subject to the Aitchisons log-ratio transform and then plotting the first two principal components. The number in parenthesis behind each age group is the number of metagenomes in the age group. Panel (**b**) shows the relative abundance distribution of the 10 most abundant bacterial families in the metagenomes within each age group, averaged over all metagenomes in the group

We divided the gut metagenomes into five age groups: infant (age 0–1), child (age 2–9), adolescent (age 10–19), adult (age 20–59) and elderly (age 60–107). In panel a of Fig. 2, the metagenomes in the infant group show a separation from the metagenomes in the other age groups. From panel b we observe that the infant metagenomes had a lower alpha diversity than the metagenomes in the other age groups since the top 10 families cover more of the total abundance, and this can also be seen in Shannon diversity plot in Supplementary Fig. S1.

**Fig. 3:**
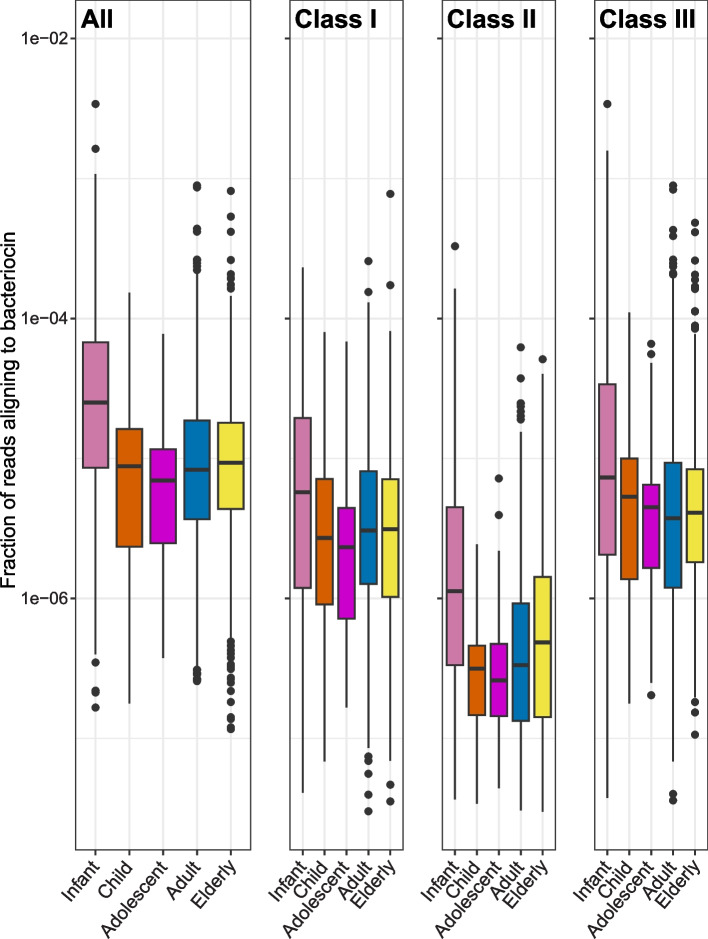
Bacteriocin gene abundance distributions in the gut metagenomes. In the leftmost panel, the total fraction of reads in each metagenome aligning to some bacteriocin is shown for the metagenomes in the infant group (pink), the child group (orange), the adolescent group (purple), the adult group (blue) and the elderly group (yellow). The median value of the infant group is 2.5 × 10^–5^ and the median value of the adult group is 8.3 × 10^–6^. Note that the y-axis is log-transformed, making differences appear smaller. The other panels are also showing the fraction of reads in each metagenome aligning to some bacteriocin, but limited to bacteriocins of class I, II or III, respectively. The difference in bacteriocin gene abundance between the infant group and each of the other age groups was significant in all cases, both when considering all matching reads and when considering reads matching bacteriocins from each of the three bacteriocin classes (*p* < 0.01)

From the bacteriocin search, we found that the fraction of bacteriocin-matching reads per total number of reads in the metagenomes of the infant group was higher compared to the metagenomes in the other age groups, as shown in Fig. 3. The same pattern was seen when comparing bacteriocin-matching reads of class I, II and III. The Wilcoxon rank-sum test indicated that the difference in abundance between the infant group and each of the other age groups was significant in all cases (*p* < 0.01), while comparisons between the other age groups yielded *p*-values above 0.05. Therefore, we treated the metagenomes of the age groups child, adolescent, adult, and elderly as one group in the downstream analyses, and defined all these metagenomes as adult-like metagenomes (*n* = 1963), while the metagenomes in the infant group from now on is specified as infant-like. A Wilcoxon rank-sum test performed for the fraction of bacteriocin-matching reads per total number of reads in the infant-like metagenomes and adult-like metagenomes indicated that there was a highly significant difference in all cases (*p* < 10^–10^).

### Enriched bacteriocin genes in infant-like metagenomes

To identify which of the bacteriocin genes detected in the bacteriocin search that were enriched in the infant-like metagenomes, the bacteriocin gene abundance in the infant-like and adult-like metagenomes were compared using a Wilcoxon rank-sum test with multiple testing correction, identifying 53 bacteriocin genes (*q* < 0.01). These belonged to 42 bacteriocin clusters based on protein sequence similarity, determined by all-versus-all pairwise alignments using blast + (similarity > 0.95). The number of infant-like metagenomes in which these 42 bacteriocin genes were detected ranged between 10 and 53%. We therefore categorized these bacteriocin genes as highly prevalent bacteriocin genes (> 30%), medium prevalent bacteriocin genes (20–30%), and low prevalent bacteriocin genes (10–20%). The highly prevalent bacteriocin genes were the encoding genes of the following bacteriocins, presented in descending prevalence: BlpU, Colicin E9, Pyocin S1, BlpK, BlpD/Thermophilin 9, Colicin and Enterolysin A. The medium prevalent bacteriocin genes were the encoding genes of the following bacteriocins: Colicin Ia, Enterolysin A, BlpU, Salivaricin 9 and rSAM-modified RiPP 019. Notice that although some of the protein names of the medium prevalent bacteriocins genes resemble some of the highly prevalent ones, their protein sequences share less than 0.95 similarity. The low prevalent bacteriocin genes were not analyzed further. Bacteriocin gene prevalence, abundance, standard deviation, and *q*-value are shown for all the enriched and clustered bacteriocins in Supplementary Table S1.

### Distribution of enriched bacteriocin genes in gut bacterial genomes

**Fig. 4:**
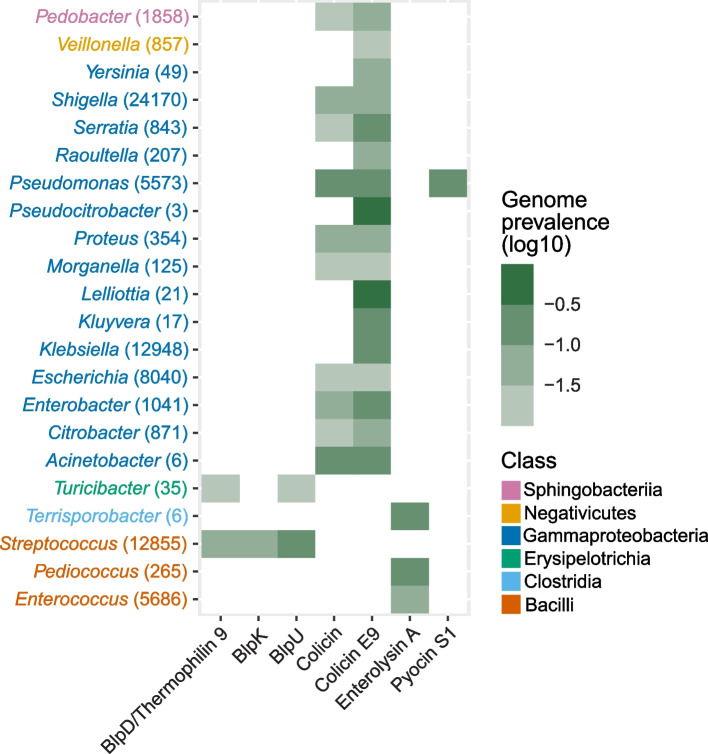
Bacteriocin gene distribution in gut bacterial genomes. The figure shows the average prevalence of the highly prevalent bacteriocin genes detected in known human gut bacterial genomes at genus-level. The number in parenthesis behind each genus is the number of genomes scanned for that genus. The color on the genus name indicates which taxonomic class the genus belongs to

Bacteriocin screening of the metagenomes identified which bacteriocin genes that were present in the gut microbiomes, and we were interested in linking the genes to their producer species in the gut. To obtain an overview of the distribution of the enriched bacteriocin genes in gut bacteria, a bacteriocin search (diamond blastx) was performed on 381 000 gut bacterial genomes [33]. For a given taxonomic rank (species or genus), a bacteriocin would typically align with a fraction of the genomes under some taxon. The average fractions within selected genera (average fractions > 0.01) shown in Fig. 4, indicate that the highly prevalent enriched bacteriocin genes are present in genomes belonging to bacteria found in the gut. The Pyocin S1-encoding gene was detected in just one genus, namely *Pseudomonas.* The Blp-encoding genes were found in two genera, both within the Firmicutes, showing the highest prevalence in *Streptococcus*. The gene encoding Enterolysin A was restricted to the Firmicutes, showing high prevalence in *Enterococcus* and *Pediococcus*. The genes encoding Colicin and Colicin E9 were detected in 10 and 17 genera, respectively, being most prevalent in Gammaproteobacteria, but they were also found in Sphingobacteriia and Negativicutes. The distribution of the highly prevalent genes among the different taxa at species-level varied, as some were restricted to specific species while others were detected in most species within one genus (Supplementary Fig. S2). The medium prevalent BlpU-encoding gene and Salivaricin 9-encoding gene displayed similar detection patterns as the highly prevalent Blp-encoding genes, showing the highest prevalence in *Streptococcus*. Compared to the highly prevalent Colicin-encoding genes, the detection of the medium prevalent Colicin Ia-encoding gene was restricted to *Shigella*, *Escherichia* and *Klebsiella*. The medium prevalent Enterolysin A-encoding gene showed a similar detection pattern as the highly prevalent Enterolysin A-encoding gene. However, the medium prevalent rSAM-modified Ripp 019 was detected in different genera then the other mentioned bacteriocins, being most prevalent in *Tannerella* and *Phocaeicola* (Supplementary Fig. S3)*.*

### Search for bacteriocin associated genes in bacteriocin containing contigs

To approach the question of functional bacteriocin production and secretion by the bacteria in the infant gut, we looked for bacteriocin associated genes adjacent to the enriched bacteriocin genes. Firstly, we assembled contigs from selected infant-like metagenomes. Next, 13 contigs that contained highly prevalent enriched bacteriocin genes were identified based on a tblastn search. The number of contigs aligning with one of the bacteriocins differed from one to three contigs, and in the case of the Blp bacteriocins, all these bacteriocins aligned to the same contig on the same location. Therefore, these genes were treated as one group in this analysis. Lastly, excerpts were made of maximum 10 000 bp upstream and downstream from the bacteriocin genes, and using blastp, genes involved in bacteriocin secretion, immunity or the bacteriocin itself, were detected. Table 1 shows that we detected (E-value < 10^–5^) both an immunity gene and a secretion protein in the proximity of the bacteriocin structural gene, indicating working operons. The taxonomic classification of the contigs concurred with the genera standing out as bacteriocin producers in the bacteriocin screening of the gut bacterial genomes (Table 1). The detected genes are illustrated on their respective contigs in Supplementary Fig. S4.

**Table 1:** Bacteriocin-related genes adjacent to highly prevalent enriched bacteriocin genes on contigs, and contig taxonomy. The table shows blastp results of two contigs from two different metagenomes per bacteriocin. A gene hit is indicated with a plus sign ( +), and an asterisk (*) indicates that the gene was not expected to be found. The different metagenomes used for assembly and the taxonomic classification of the contigs are included in the table

Bacteriocin^a^	Secretion protein gene^bc^	Immunity gene^c^	Bacteriocin gene^c^	Metagenomes for assembly^d^	Taxonomic classification of contig
**Blp bacteriocins**	+	+	+	Metagenome A	*Streptococcus* sp. C150
	+	+	+	Metagenome B	*Streptococcus vestibularis*
**Colicin**	+	+	+	Metagenome D	*Klebsiella aerogenes*
	+	+	+	Metagenome E	*Klebsiella aerogenes*
**Enterolysin A**	*	*	+	Metagenome F	*Enterococcus faecalis*
	*	*	+	Metagenome G	*Enterococcus faecalis*

^a^Genes of these bacteriocins were detected on contigs, and the same contigs yielded results with blastp as shown in this table

^b^Secretion proteins refers to ABC transporter for Blp bacteriocins and colicin lysis protein for Colicin. Enterolysin A does not have a known secretion protein and immunity gene

^c^All hits had an E-value < 10^–5^. The positive hits the Blp bacteriocins and Colicin are located on the same contig

^d^Infant-like metagenome with the highest and second highest detected abundance of the bacteriocin gene, respectively. Colicin, Colicin E9 and Pyocin S1 were detected on the same position on the same contig from Metagenome D, and the blastp results from this contig are represented by Colicin in this table

### Association between bacteriocin genes and within-species diversity in the infant gut

**Fig. 5:**
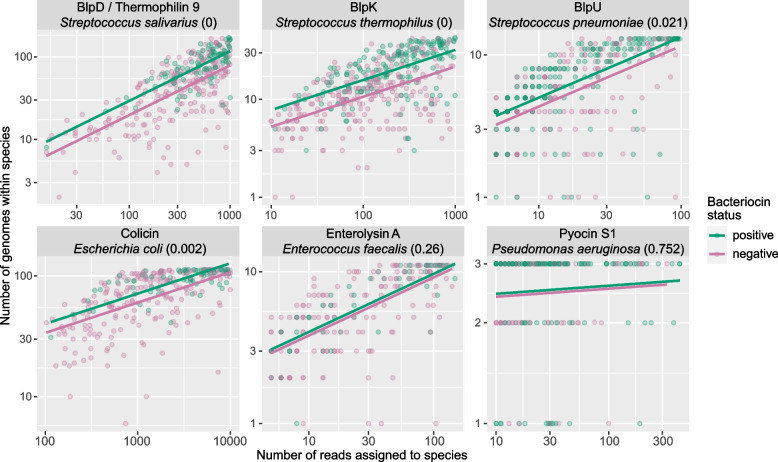
Diversity is affected by bacteriocin presence/absence. Each dot is a metagenome with or without the corresponding bacteriocin (colors). The number of genomes detected (y-axis) reflects the diversity within a species at a given abundance, here quantified as the number of reads (x-axis) after all metagenomes were rarefied to the same total number of reads (1 million). The lines are regression lines, and the *p*-values in parentheses indicate if there is a significant difference in levels of the regression lines

To assess the effects of bacteriocins on the bacterial composition in the infant gut, we investigated the within-species diversity in the infant-like metagenomes, focusing on the enriched bacteriocin genes and the producer species of these. The reason for looking at within-species diversity is that bacteriocins are known to mostly affect close relatives. The producer species were chosen based on the bacteriocin search in gut bacterial genomes, selecting the species with the highest bacteriocin gene prevalence. We estimated within-species diversity for a given metagenome by classifying metagenome reads and determining the number of different genomes from the species in which the reads were assigned. A higher number of genomes indicates a higher within-species diversity, but this value depends on how abundant the species is in the metagenome. Therefore, we also collected the number of reads per 1 million that were assigned to the species. In Fig. 5, the relation between the number of genomes (y-axis) and the number of reads assigned to the same species (x-axis) is shown. But more important, when coloring metagenomes according to bacteriocin gene presence or absence, we observe a pattern. Metagenomes with a bacteriocin gene consistently exhibited a larger diversity, i.e. more genomes per species. This difference is significant in most cases, and in the remaining cases, the green regression line is always (slightly) above the pink, indicating the same trend. The case for the Pyocin S1-encoding gene and *Pseudomonas aeruginosa* (lower right panel) is not significant, likely because there were only three HumGut genomes for this species, and therefore the resolution became too low. A more extensive list of producer species was analyzed, and the same trend can be seen for these species as well (Supplementary Fig. S5). The medium prevalent bacteriocin genes display similar regression trends as the highly prevalent genes (Supplementary Fig. S5).

## Discussion

The bacteriocin gene enrichment in infants compared to adults can possibly be linked to active competition between early colonizers in the gut. Another body site enriched in bacteriocins is the oral cavity [21, 34, 35], which in similarity to the infant gut is inhabited by founder communities. Both habitats have fluctuations in the environmental conditions, leading to continual recolonization of the habitats [36–38]. However, the causal association between bacteriocins and founder communities remains to be determined.

The association between bacteriocin gene presence and increased strain diversity in the infant gut for bacteriocin-associated species was significant for the highly prevalent Colicin-encoding gene. Considering that Colicin is a bacteriocin with a narrow spectrum of activity [39], our finding indicates that intraspecies competition driven by bacteriocins can promote strain diversity. The same was seen for several of the Blp-encoding genes, and although the knowledge on the activity spectra of Blp bacteriocins is limited, it has been reported that four different Blp peptides compose the multipeptide bacteriocin Thermophilin 9 [40, 41], which has a narrow activity spectrum. The broad spectrum bacteriocin Enterolysin A [42, 43], however, did not display a difference in strain diversity in the metagenomes depending on bacteriocin detection, indicating that the inhibition spectrum of bacteriocins could be important for within-species diversity.

The finding of an association between bacteriocin gene presence and increased strain diversity might be explained by intransitive bacterial competition. As reported by Kerr et al. [9] and elaborated further by Abrudan, Brown, and Rozen [10], the presence of bacteriocin-producing strains may very well lead to an increased diversity. When three strains that compete for the same niche have one of the following traits each; bacteriocin producer and resistance, bacteriocin resistance only or bacteriocin sensitive but without the cost of neither production nor resistance, intransitive competition dynamics in form of the RPS model may occur [9, 10]. Assuming that the strains are counterbalanced in an RPS manner, the complexity of the bacterial community can be expanded by adding new bacteriocin producers and strains that are resistant and sensitive to the new bacteriocins [10]. In this study, the within-species diversity in the representation of reads matching that species, was consistently larger for the bacteriocin-containing metagenomes compared to those without. Thus, bacteriocin gene presence was found to be associated to increased strain diversity within the producer species, and this can possibly be enabled by RPS dynamics. As for the bacteriocin enrichment, experimental evidence is needed to determine if intransitive bacterial competition is an explanation for the strain diversification in the infant gut.

Regarding the highly prevalent bacteriocin genes, the presence of Blp-encoding genes in gut bacterial genomes was predominantly observed in genera belonging to the Firmicutes, being most prevalent in *Streptococcus*. This genus is known for production of these bacteriocins [44, 45]. We identified the Enterolysin A-encoding gene in genera restricted to the Firmicutes, and the highest prevalence of this bacteriocin was in *Enterococcus,* which is in line with previous observations [42, 46]. We found the Pyocin S1-encoding gene in just one genus, *Pseudomonas*, suggesting that *Pseudomonas* is the only producer of this bacteriocin. However, the colicin-encoding genes, which are known to be produced by *Escherichia coli* and some close relatives [39], were found to be abundant in *Pseudomonas*. Homology between colicins and pyocins has been characterized previously [47]. The wide distribution of colicin-encoding genes in different genera belonging to Proteobacteria may indicate that these genes can be disseminated by horizontal gene transfer of colicin-encoding plasmids [48]. The classification of metagenome-assembled contigs that contained the encoding genes of the Blp bacteriocins, Colicin and Enterolysin A ties well with the bacteriocin-associated genera discussed.

The detection of genes related to bacteriocin secretion and immunity adjacent to bacteriocin genes on Blp- and Colicin-associated contigs indicates that the infant gut inhabits potential bacteriocin producers. The secretion protein gene detected for the Blp bacteriocins was the ABC transporter, a protein required for bacteriocin maturation and secretion [49]. For Colicin, the gene encoding colicin lysis protein was detected, a protein involved in cell release of group A colicins, preventing the colicins from accumulating in the cytoplasm [39]. No secretion proteins or immunity proteins are described for Enterolysin A [50]. The illustrations of the detected Blp and Colicin gene clusters (Fig. S4) do not resemble known gene clusters for these bacteriocin groups [51–54]. However, among the different gene clusters described for these groups, there does not seem to be a consensus on how the genes are structured [44, 55], indicating that the gene cluster structure of these bacteriocins may be diverse.

It is clear that an in silico search for bacteriocin genes in metagenomes will also lead to a number of false positive matches. Even if open reading frames with a sequence matching a large part of a known bacteriocin gene are found, the verification of a truly functional operon requires more extensive studies. The results we present in Table 1 and Supplementary Fig. S4 are merely indicators of this.

## Conclusions

In summary, the finding of enriched bacteriocin genes in the infant gut in this study suggests that bacteriocins conceivably are of significance in the early stages of human gut microbiota establishment. We have verified that the members of the infant microbiome harbor bacteriocin loci with essential genes for secretion and immunity, which substantiate their potential of bacteriocin production. Based on two previously suggested bottom-up selection theories, we hypothesized that bacteriocins can affect the strain diversity of the human gut microbiota. Our results support that bacteriocins could promote strain diversity in the infant gut microbiota, possibly enabled by intransitive competition. Yet, further work is necessary to study the bacteriocin-mediated interactions between strains in an experimental setup and identify possible mechanisms for intransitive competition.

## Methods

The methods are described following the same structure as given in Fig. 1.

### Bacteriocin screening of gut metagenomes

#### Data

From all available BioProjects at NCBI/SRA [56] a collection of human gut metagenomes from healthy individuals was downloaded. Healthy individuals are in general the healthy controls in research projects, as described by Hiseni et al. [33]. From these, we collected all metagenomes with information about the persons’ age, 2564 metagenomes in total. The number of metagenomes per age can be found in Supplementary Fig. S6. Next, all bacteriocin protein sequences in the bacteriocin databases BAGEL4 [30] and BACTIBASE [31, 32], comprising 1231 sequences, were collected.

#### Taxonomic profiles

For each of the metagenomes, the taxonomic composition at the species-level was estimated, using the kraken2 [57, 58] and bracken [59] software, and using the HumGut genome collection as a reference database [33]. The metagenomes were grouped into five age groups to explore presence of bacteriocins in different stages in life: infant (age 0–1), child (age 2–9), adolescent (age 10–19), adult (age 20–59) and elderly (age 60–107). Beta diversity was computed from the taxonomic profiles, by first using the Aitchisons log-ratio transform [60] and then a Principal Component Analysis of the resulting profile matrix, as seen in panel a of Fig. 2. The Shannon diversity for samples at each age showed that persons of age 0 and 1 year had substantially lower diversity than those of 2 years and above, which is as expected [61]. See Supplementary Fig. S1 for details on this. Based on the similarities between the metagenomes in the child, adolescent, adult and elderly groups, these metagenomes were later grouped together and referred to as the adult-like metagenomes in the rest of the article. The infant group was kept, and in the rest of the article the metagenomes in this group is referred to as the infant-like metagenomes.

#### Sequence similarity search

All unique bacteriocin protein sequences, 1075 sequences, were used to build a diamond database [62]. For each metagenome, all the reads were searched against the database of bacteriocin proteins, using the diamond blastx. This resulted in a set of bacteriocin-matching reads for each metagenome. As an estimate for the abundance of bacteriocin genes in a persons’ gut, the number of unique reads that gave at least one match against any bacteriocin was counted for each metagenome. The diamond results were processed, filtering out all hits having an E-value above 10^–5^. The number of unique matching reads was then divided by the total number of reads for the metagenome, to obtain the bacteriocin gene abundance for each metagenome. The downstream analysis focuses on the bacteriocin genes that are enriched in infants, i.e. they tend to have a higher bacteriocin gene abundance in infant-like metagenomes than in adult-like metagenomes. This enrichment was found by using a Wilcoxon rank-sum test for each bacteriocin, testing if the bacteriocin gene abundance has a larger expected value in infant-like metagenomes than in adult-like metagenomes. Since this test was performed for each of the bacteriocins separately, the multiple testing was corrected for by converting all *p*-values to *q*-values, controlling the False Discovery Rate [63]. This resulted in a ranked list of enriched bacteriocin genes, ranked by *q*-value. It must be noted that some bacteriocin genes with a very small q-value was found present (abundance > 0) in only a very small number of metagenomes from both infant- and adult-like metagenomes. To eliminate such cases, only bacteriocins present in at least 10% of the infant-like metagenomes were considered.

#### Bacteriocin grouping

Up to this point there had been made no attempt to group the bacteriocins, even if it was apparent from the start that several entries in the bacteriocin databases are variants of the same protein. However, the enriched bacteriocins were now grouped based on their sequence similarity. An all-versus-all pairwise alignments using blast + was performed [64] and the bit-score for each alignment was collected. The similarity of sequence *A* and *B* was then computed as the *2S(A,B)/(S(A,A)* + *S(B,B))* where *S(A,B)* is the bit-score of the alignment between *A* and *B*, and *S(A,A)* and *S(B,B)* are the corresponding self-alignment bit-scores. If *A* and *B* are identical this becomes 1.0. Pairs having a similarity of at least 0.95 were clustered as one bacteriocin.

### Bacteriocin screening of genomes

Having the set of enriched and clustered bacteriocins, these were associated to some bacterial taxa in the human infant gut. Again, a diamond search was performed against the bacteriocins, this time using the HumGut genome collection instead of metagenome reads as queries. The HumGut collection contains roughly 30 000 genomes, where each represents a group of very similar genomes, in total around 381 000 genomes. All these genomes were used in the search, and from these results a taxonomic profile for each bacteriocin was computed, where the value for a given bacteriocin and taxon is the fraction of genomes from that taxon having some match against the bacteriocin. Thus, for each enriched and clustered bacteriocin the associated taxa are those with the largest values in this profile.

### Within-species diversity analysis

For a given bacteriocin and its associated species, the within-species alpha-diversity across infant-like metagenomes was investigated. Infant-like metagenomes were categorized as either bacteriocin positive or negative, reflecting if the given bacteriocin was detected or not. In our HumGut genome collection there are typically several genomes within each species since genomes are clustered at 97.5% Average Nucleotide Identity. A custom kraken2 database was made where reads are either assigned to such a genome or not assigned at all. Then all the reads in all the infant-like metagenomes were classified using this database. The metagenomes were rarefied to 1 million reads. For each metagenome, positive or negative, the number of genomes having reads assigned was counted, as a proxy for strain diversity. The total number of reads within the species was also computed. Thus, for each infant-like metagenome, the total number of reads associated with a species, as well as how many genomes that was detected from that species was obtained.

### Assembly and blasting of contigs

To verify that a metagenome where many reads have bacteriocin hits actually contained the bacteriocin gene, as well as looking for evidence of a truly functional operon, assembly of some of the metagenomes were done. For each bacteriocin, the infant-like metagenomes with the two highest number of hits were chosen for assembly. In total, 8 distinct metagenomes, containing 7.9 × 10^6^ -3.8 × 10^8^ read pairs, were assembled to contigs using metaspades [65]. The contigs were screened for their corresponding bacteriocin gene using tblastn. From each contig with hits, the surrounding region was extracted, and all Open Reading Frames (ORFs) were extracted from the selected region. Then the ORFs were translated, and a blastp search was done against the full nr-database at NCBI to see if they contained genes required for a functional bacteriocin (e.g. immunity genes and secretion protein genes). The contigs were taxonomically classified using the kraken2 software and the HumGut genome collection as a reference database.

### Statistics and reproducibility

The metagenomes analyzed in this study are all publicly available at NCBI/SRA [56].

As described above, the non-parametric Wilcoxon test was used to test for differing bacteriocin gene abundance between infants- and adult-like metagenomes, followed by a correction for multiple testing.

The difference in within-species diversity presented in Fig. 5 was analyzed by a simple analysis of covariance model

$$y_{\mathit i}=b_{\mathit0}+a_{\mathit0}l_i+b_{\mathit1}x_i+e_i$$

where the response *y*_*i*_ is the number of genomes detected within the species for metagenome *i*, the explanatory variable *x*_*i*_ is the abundance of the species (reads per 1 million reads) for metagenome *i,* the indicator variable *I*_*i*_ is 1 if metagenome *i* has bacteriocin and 0 if not and *e*_*i*_ is the random error term. The interesting parameter is *a*_*0*_. If this is > 0 it means there is a significant increase in diversity between bacteriocin and non-bacteriocin metagenomes, regardless of how abundant the species is in the metagenomes. The *p*-values in Fig. 5 refers to the testing of *a*_*0*_ = *0* versus *a*_*0*_ is nonzero.

## Supplementary Information

**Additional file 1: Figure S1.** Shannon diversity of the metagenomes in this study.

**Additional file 2: Table S1.** Prevalence and abundance of the enriched and clustered bacteriocin genes.

**Additional file 3: Figure S2.** Bacteriocin gene distribution in gut bacterial genomes at species-level.

**Additional file 4: Figure S3.** Distribution of highly prevalent and medium prevalent bacteriocin genes in gut bacterial genomes.

**Additional file 5: Figure S4.** Distribution of bacteriocin associated genes on contigs.

**Additional file 6: Figure S5.** Within-species diversity analysis of highly prevalent and medium prevalent enriched bacteriocin genes.

**Additional file 7: Figure S6.** Age distribution of metagenomes.

**Additional file 8:** Metagenome accession numbers.

## Data Availability

The accession numbers of the metagenomes analyzed during the current study are listed in Additional file 8 and all the metagenomes can be found at NCBI/SRA (https://www.ncbi.nlm.nih.gov/sra/).
