# Supplementary material for: Metagenome-mining indicates an association between bacteriocin presence and strain diversity in the infant gut
Source: BMC Genomics. 2023 May 31;24:295. doi: 10.1186/s12864-023-09388-0 (PMC10230729; doi:10.1186/s12864-023-09388-0)
Supplement: Supplementary file 1 — Additional file 1: Figure S1. Shannon diversity of the metagenomes in this study. [file 12864_2023_9388_MOESM1_ESM.docx]

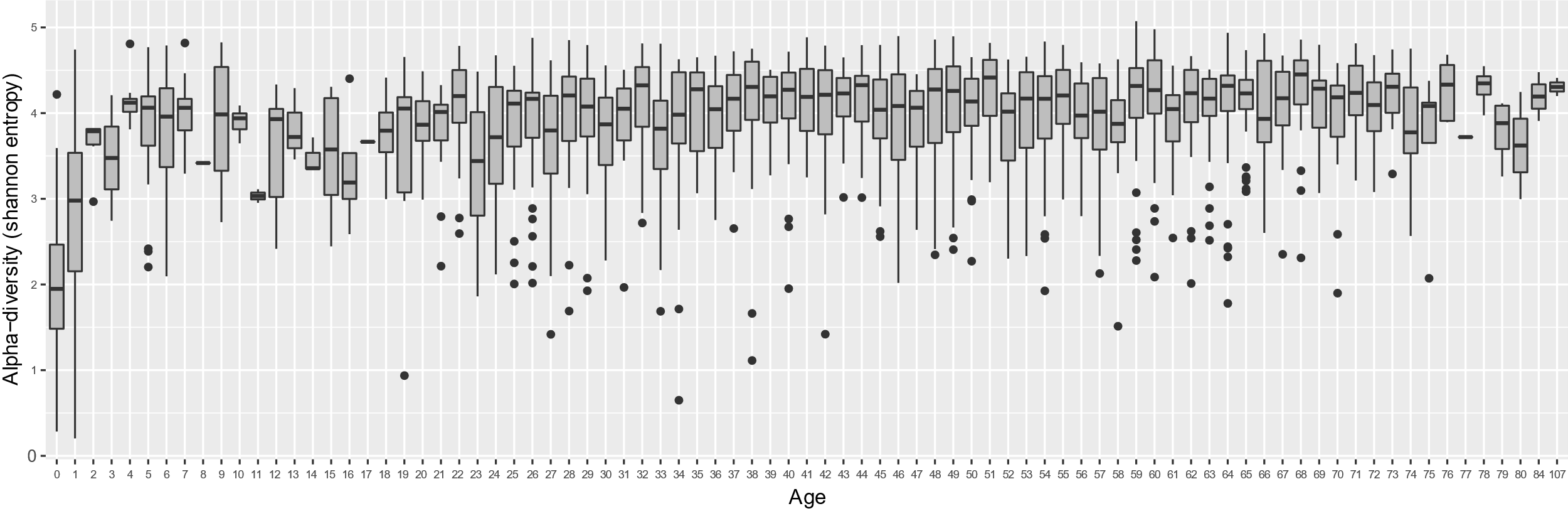


**Figure S1: Shannon diversity of the metagenomes in this study.** The figure shows Shannon entropy measures of the metagenomes per age category. The metagenomes in age category 0 and 1 stand out due to their low bacterial diversity compared to the other age categories. The largest difference in Shannon diversity was between age category 0 and 1 and the rest (Wilcoxon rank-sum test,
p < 10^-159^).
