# Supplementary material for: Metagenome-mining indicates an association between bacteriocin presence and strain diversity in the infant gut
Source: BMC Genomics. 2023 May 31;24:295. doi: 10.1186/s12864-023-09388-0 (PMC10230729; doi:10.1186/s12864-023-09388-0)
Supplement: Supplementary file 2 — Additional file 2: Table S1. Prevalence and abundance of the enriched and clustered bacteriocin genes. [file 12864_2023_9388_MOESM2_ESM.pdf]

**Table S1: Prevalence and abundance of the enriched and clustered bacteriocin genes.** The 42 enriched and clustered bacteriocin genes are listed in the table, together with their prevalence and abundance in the infant and adult groups. The corresponding p-values and q-values are given. The unique bacteriocin ID was given to each unique bacteriocin sequence to keep them apart.

| Unique bacteriocin ID | Name | Prevalence infant | Prevalence adult | Abundance infant | Abundance adult | Abundance difference | p-value | q-value |
|---|---|---|---|---|---|---|---|---|
| Bac809 | BlpU | 0.53 | 0.36 | 1.78E-06 | 2.72E-07 | 1.51E-06 | 3.53E-24 | 7.86E-23 |
| Bac957 | Colicin_E9 | 0.52 | 0.29 | 5.18E-06 | 8.73E-07 | 4.31E-06 | 1.38E-41 | 1.99E-39 |
| Bac1041 | Pyocin S1 | 0.51 | 0.18 | 8.66E-06 | 1.94E-07 | 8.46E-06 | 4.87E-77 | 2.82E-74 |
| Bac682 | BlpK | 0.51 | 0.30 | 1.63E-06 | 1.92E-07 | 1.44E-06 | 1.47E-31 | 8.32E-30 |
| Bac935 | BlpD / Thermophilin 9 | 0.49 | 0.27 | 1.46E-06 | 1.85E-07 | 1.28E-06 | 3.35E-33 | 3.24E-31 |
| Bac948 | Colicin | 0.44 | 0.26 | 2.63E-06 | 8.30E-07 | 1.80E-06 | 1.44E-26 | 3.78E-25 |
| Bac1009 | Enterolysin A | 0.32 | 0.19 | 7.47E-06 | 1.71E-07 | 7.30E-06 | 5.71E-15 | 8.93E-14 |
| Bac1057 | Colicin-Ia | 0.28 | 0.24 | 1.34E-05 | 2.66E-06 | 1.07E-05 | 3.03E-03 | 8.16E-03 |
| Bac978 | enterolysin_A | 0.27 | 0.13 | 5.92E-06 | 6.81E-08 | 5.85E-06 | 1.19E-20 | 2.23E-19 |
| Bac808 | BlpU | 0.25 | 0.11 | 1.79E-07 | 2.86E-08 | 1.51E-07 | 1.97E-18 | 3.45E-17 |
| Bac364 | Salivaricin_9 | 0.24 | 0.13 | 6.47E-07 | 1.03E-07 | 5.44E-07 | 9.25E-12 | 9.23E-11 |
| Bac577 | rSAM-modified_RiPP_019 | 0.21 | 0.31 | 2.30E-07 | 2.29E-07 | 9.81E-10 | 5.35E-06 | 2.58E-05 |
| Bac339 | Salivaricin_G32 | 0.19 | 0.14 | 8.03E-07 | 1.42E-07 | 6.61E-07 | 3.60E-04 | 1.27E-03 |
| Bac336 | Streptococcin_A-FF22 | 0.19 | 0.13 | 5.74E-07 | 1.16E-07 | 4.58E-07 | 2.37E-05 | 1.02E-04 |
| Bac945 | Carocin_D | 0.18 | 0.05 | 4.05E-06 | 3.36E-08 | 4.01E-06 | 7.35E-28 | 2.50E-26 |
| Bac858 | UviB | 0.18 | 0.14 | 5.21E-07 | 1.09E-07 | 4.12E-07 | 9.98E-04 | 3.12E-03 |
| Bac344 | Ruminococcin_A | 0.18 | 0.13 | 1.17E-06 | 2.04E-07 | 9.71E-07 | 9.53E-05 | 3.68E-04 |
| Bac353 | Butyrivibriocin_OR79 | 0.18 | 0.13 | 1.15E-06 | 1.79E-07 | 9.68E-07 | 2.11E-04 | 7.73E-04 |
| Bac357 | Butyrivibriocin | 0.18 | 0.11 | 1.04E-06 | 1.73E-07 | 8.63E-07 | 1.14E-05 | 5.28E-05 |
| Bac348 | BLD_1648 | 0.17 | 0.11 | 4.61E-06 | 3.63E-07 | 4.25E-06 | 1.06E-06 | 5.74E-06 |
| Bac346 | Variacin | 0.16 | 0.10 | 7.85E-07 | 1.14E-07 | 6.70E-07 | 4.67E-05 | 1.90E-04 |
| Bac652 | rSAM-modified_RiPP_094 | 0.15 | 0.26 | 2.44E-07 | 2.29E-07 | 1.55E-08 | 3.66E-07 | 2.23E-06 |
| Bac939 | alveicin_B_Bacteriocintoxin | 0.15 | 0.09 | 1.15E-06 | 1.90E-07 | 9.62E-07 | 2.94E-07 | 1.81E-06 |
| Bac582 | rSAM-modified_RiPP_024 | 0.15 | 0.27 | 3.81E-07 | 2.37E-07 | 1.44E-07 | 2.04E-07 | 1.29E-06 |
| Bac968 | Colicin-A | 0.15 | 0.08 | 7.28E-06 | 3.22E-07 | 6.96E-06 | 1.05E-06 | 5.72E-06 |

| | | | | | | | | |
|---|---|---|---|---|---|---|---|---|
| Bac967 | Colicin-10 | 0.15 | 0.25 | 4.80E-06 | 1.45E-06 | 3.35E-06 | 1.78E-06 | 9.29E-06 |
| Bac581 | rSAM-modified_RiPP_023 | 0.14 | 0.26 | 3.63E-07 | 3.15E-07 | 4.79E-08 | 3.04E-09 | 2.44E-08 |
| Bac324 | Pneumolancidin PldA1 | 0.14 | 0.09 | 1.36E-07 | 3.86E-08 | 9.70E-08 | 1.24E-04 | 4.69E-04 |
| Bac810 | Bovicin_255_peptide | 0.14 | 0.09 | 1.13E-07 | 8.91E-08 | 2.39E-08 | 4.34E-04 | 1.50E-03 |
| Bac573 | rSAM-modified_RiPP_015 | 0.14 | 0.21 | 1.77E-07 | 7.34E-08 | 1.04E-07 | 8.26E-04 | 2.64E-03 |
| Bac987 | klebicin_C_activity | 0.13 | 0.05 | 3.46E-07 | 5.29E-08 | 2.93E-07 | 1.69E-11 | 1.63E-10 |
| Bac1037 | Colicin-V (Microcin-V) | 0.13 | 0.07 | 1.52E-06 | 7.39E-08 | 1.45E-06 | 4.25E-07 | 2.57E-06 |
| Bac496 | Streptide | 0.12 | 0.08 | 5.78E-08 | 2.34E-08 | 3.44E-08 | 7.75E-04 | 2.52E-03 |
| Bac328 | Pneumolancidin PldA4 | 0.12 | 0.08 | 1.68E-07 | 2.51E-08 | 1.43E-07 | 3.16E-03 | 8.48E-03 |
| Bac503 | Microcin_M | 0.12 | 0.04 | 7.58E-07 | 4.25E-08 | 7.16E-07 | 5.59E-12 | 5.78E-11 |
| Bac976 | Colicin-N | 0.12 | 0.06 | 4.75E-06 | 9.27E-08 | 4.66E-06 | 7.49E-08 | 4.99E-07 |
| Bac988 | klebicin_C_phage_associated_protein | 0.11 | 0.05 | 2.26E-06 | 2.80E-08 | 2.24E-06 | 1.25E-08 | 9.42E-08 |
| Bac347 | Pneumolancidin PldA3 | 0.11 | 0.06 | 1.04E-07 | 1.57E-08 | 8.84E-08 | 1.87E-05 | 8.19E-05 |
| Bac363 | Pneumolancidin PldA2 | 0.11 | 0.07 | 1.17E-07 | 3.24E-08 | 8.46E-08 | 2.84E-04 | 1.02E-03 |
| Bac989 | klebicin_C_phage_associated_protein | 0.11 | 0.05 | 1.68E-06 | 2.67E-08 | 1.65E-06 | 7.26E-09 | 5.68E-08 |
| Bac883 | Delta-lysinI | 0.11 | 0 | 2.43E-07 | 0.00E+00 | 2.43E-07 | 2.89E-49 | 8.35E-47 |
| Bac502 | Microcin_H47_(MccH47) | 0.10 | 0.04 | 1.83E-07 | 2.95E-08 | 1.54E-07 | 4.25E-11 | 3.97E-10 |
