# Supplementary material for: Metagenome-mining indicates an association between bacteriocin presence and strain diversity in the infant gut
Source: BMC Genomics. 2023 May 31;24:295. doi: 10.1186/s12864-023-09388-0 (PMC10230729; doi:10.1186/s12864-023-09388-0)
Supplement: Supplementary file 3 — Additional file 3: Figure S2. Bacteriocin gene distribution in gut bacterial genomes at species-level. [file 12864_2023_9388_MOESM3_ESM.docx]

**
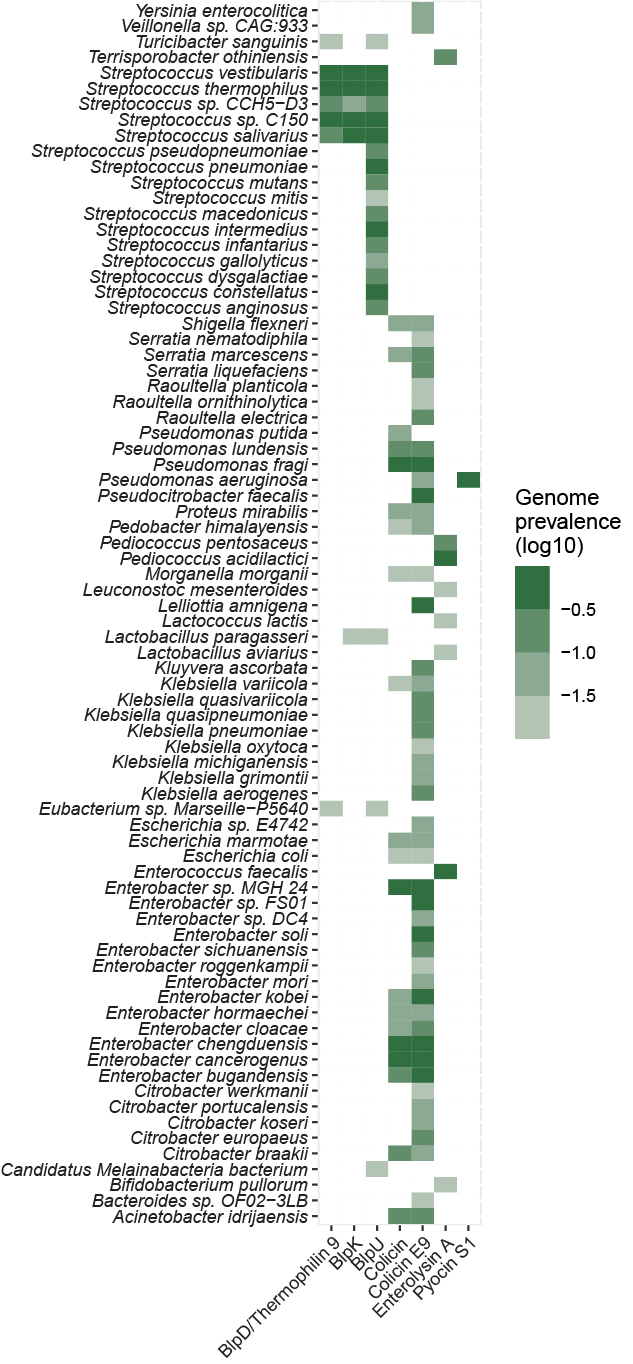
**

**Figure S2: Bacteriocin gene distribution in gut bacterial genomes at species-level.** The figure shows the prevalence of the highly prevalent bacteriocin genes detected in known human gut bacterial genomes at species-level.
