# Supplementary material for: Metagenome-mining indicates an association between bacteriocin presence and strain diversity in the infant gut
Source: BMC Genomics. 2023 May 31;24:295. doi: 10.1186/s12864-023-09388-0 (PMC10230729; doi:10.1186/s12864-023-09388-0)
Supplement: Supplementary file 4 — Additional file 4: Figure S3. Distribution of highly prevalent and medium prevalent bacteriocin genes in gut bacterial genomes. [file 12864_2023_9388_MOESM4_ESM.docx]

**
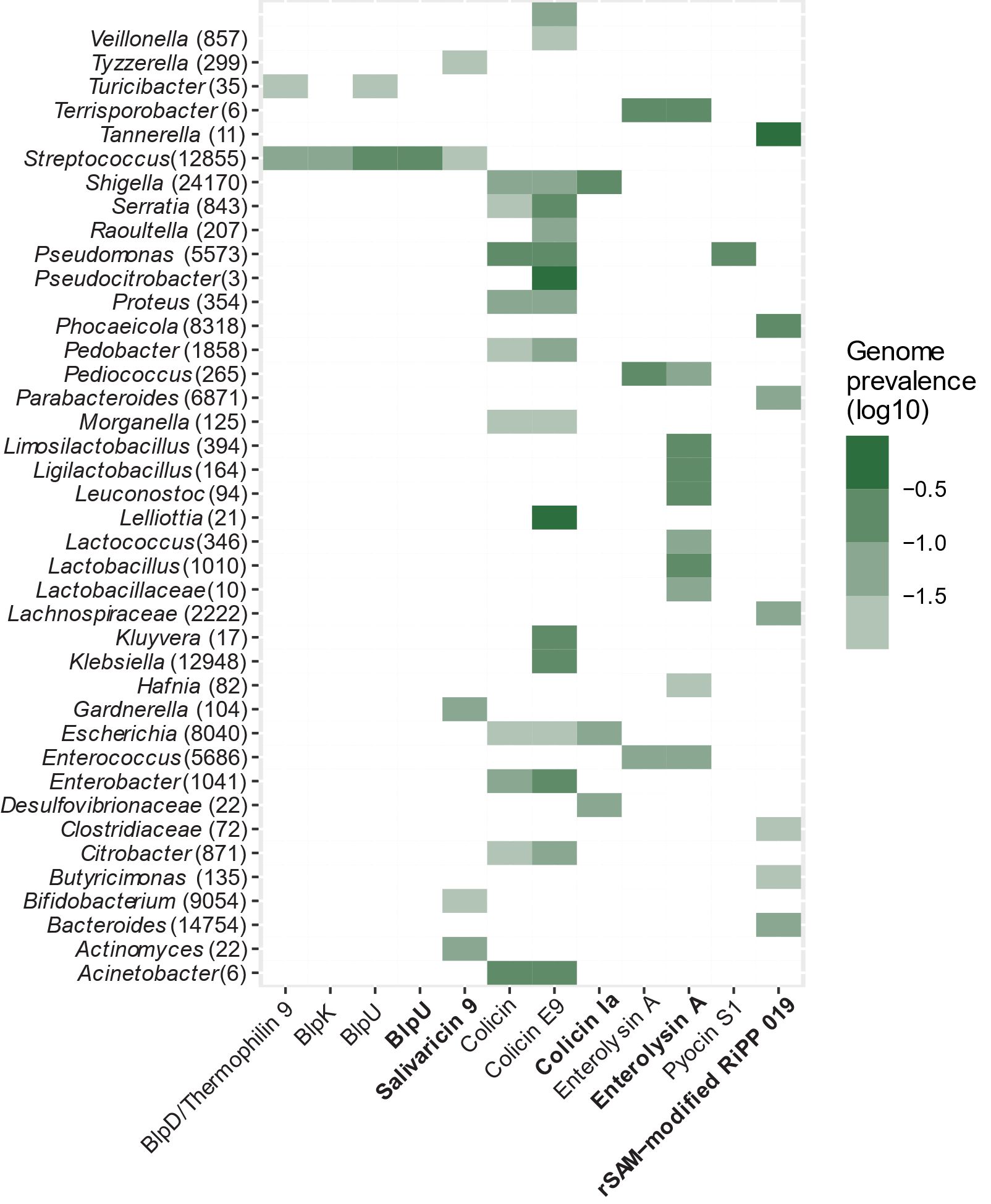
**

**Figure S3: Distribution of highly prevalent and medium prevalent bacteriocin genes in gut bacterial genomes.** The figure shows the average prevalence of the highly prevalent and medium prevalent enriched bacteriocin genes detected in known human gut bacterial genomes at genus-level. The bacteriocin names in bold are the medium prevalent bacteriocins. The number in parenthesis behind each genus is the number of genomes scanned for that genus.
