# Supplementary material for: Metagenome-mining indicates an association between bacteriocin presence and strain diversity in the infant gut
Source: BMC Genomics. 2023 May 31;24:295. doi: 10.1186/s12864-023-09388-0 (PMC10230729; doi:10.1186/s12864-023-09388-0)
Supplement: Supplementary file 5 — Additional file 5: Figure S4. Distribution of bacteriocin associated genes on contigs. [file 12864_2023_9388_MOESM5_ESM.docx]

**
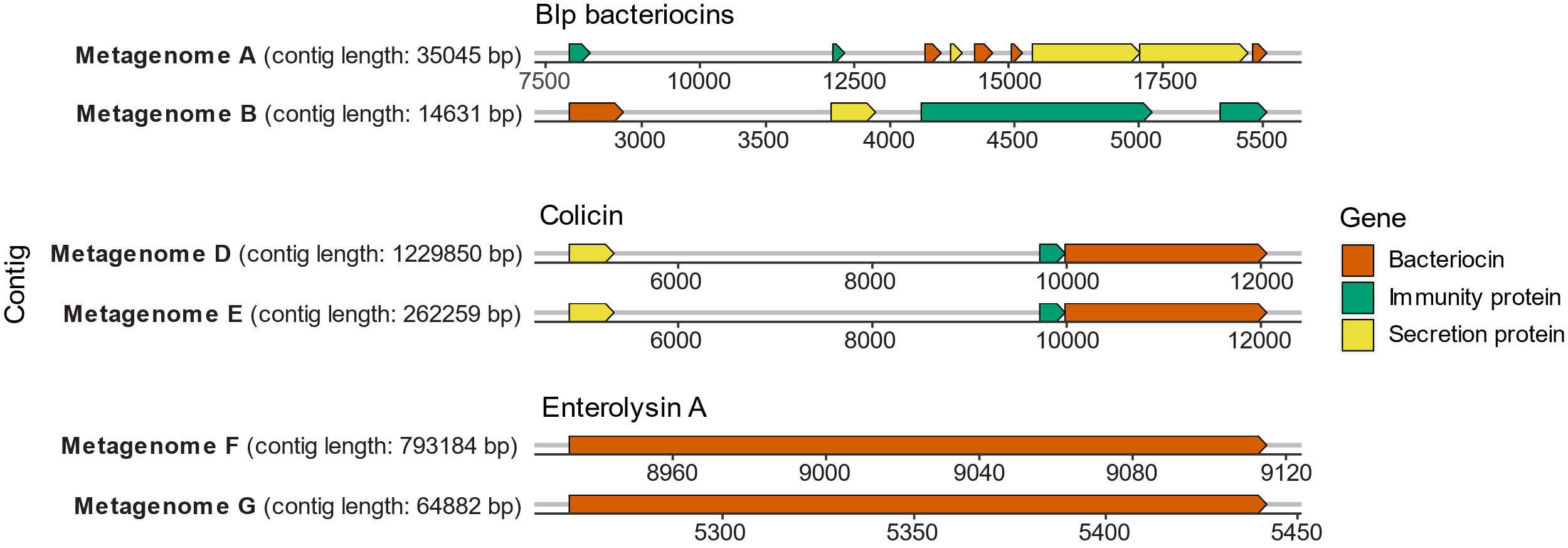
**

**Figure S4: Distribution of bacteriocin associated genes on contigs.** The figure shows how the bacteriocin structural genes (orange), immunity genes (green) and secretion protein genes (yellow) are distributed on the contig excerptions form the different metagenomes. As expected, the Enterolysin A associated contigs only contained the bacteriocin gene, but for the contigs containing Colicin and Blp bacteriocins, immunity genes and secretion protein genes were detected as well. The contig from Metagenome D aligned with Colicin, Colicin E9 and Pyocin S1, and the result is represented by Colicin in this figure.
