# Supplementary material for: Metagenome-mining indicates an association between bacteriocin presence and strain diversity in the infant gut
Source: BMC Genomics. 2023 May 31;24:295. doi: 10.1186/s12864-023-09388-0 (PMC10230729; doi:10.1186/s12864-023-09388-0)
Supplement: Supplementary file 6 — Additional file 6: Figure S5. Within-species diversity analysis of highly prevalent and medium prevalent enriched bacteriocin genes. [file 12864_2023_9388_MOESM6_ESM.docx]

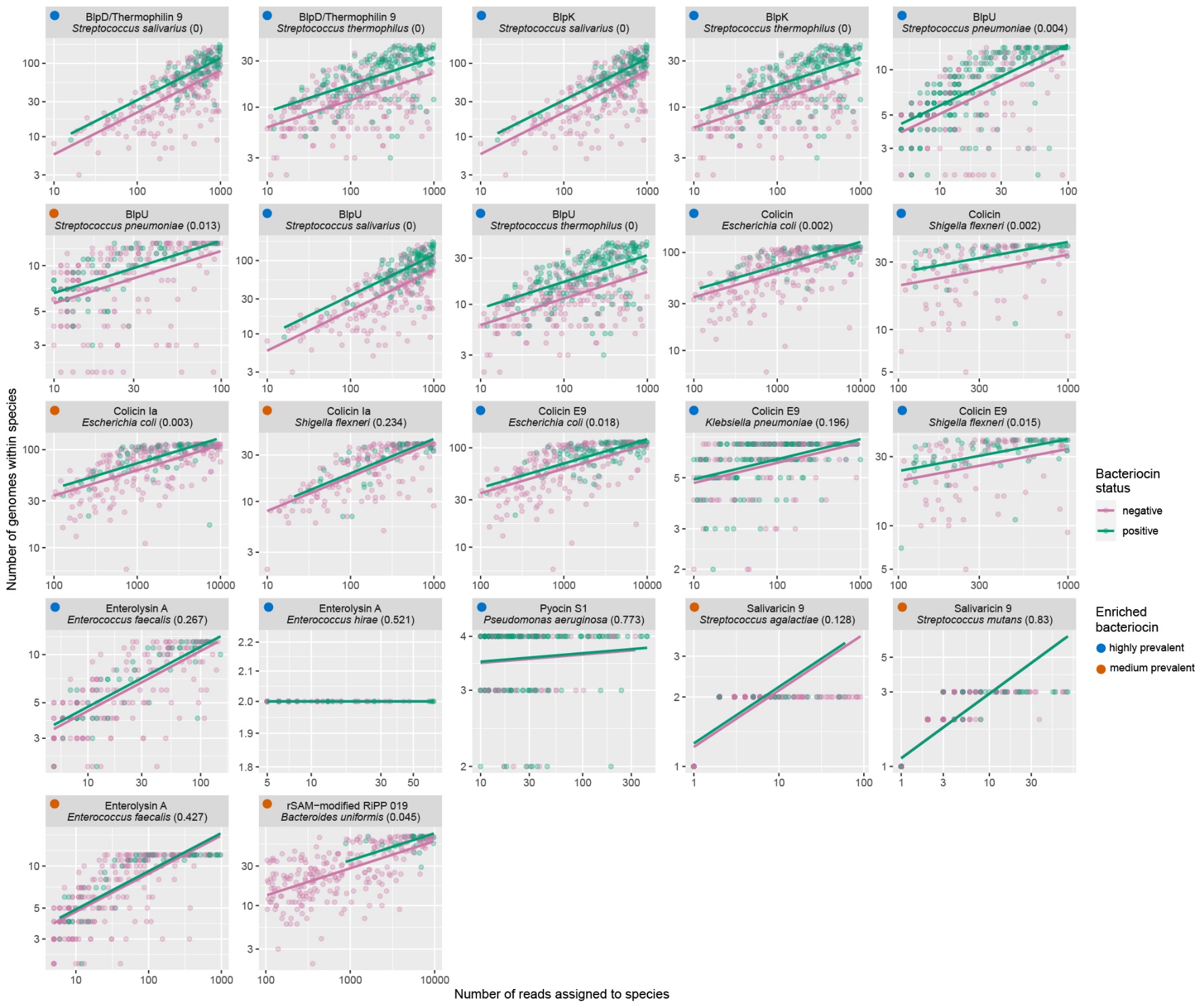
**Figure S5: Within-species diversity analysis of highly prevalent and medium prevalent enriched bacteriocin genes.** Each dot is a metagenome with or without the corresponding bacteriocin (colors). The number of genomes detected (y-axis) reflects the diversity within a species at a given abundance, here quantified as the number of reads (x-axis) after all metagenomes were rarefied to the same total number of reads (1 million). The random selection of reads during the rarefaction results in slightly different outcomes each rerun. The lines are regression lines, and the p-values in parentheses indicate if there is a significant difference in levels of the regression lines.
