# Supplementary material for: Metagenome-mining indicates an association between bacteriocin presence and strain diversity in the infant gut
Source: BMC Genomics. 2023 May 31;24:295. doi: 10.1186/s12864-023-09388-0 (PMC10230729; doi:10.1186/s12864-023-09388-0)
Supplement: Supplementary file 7 — Additional file 7: Figure S6. Age distribution of metagenomes. [file 12864_2023_9388_MOESM7_ESM.docx]

**
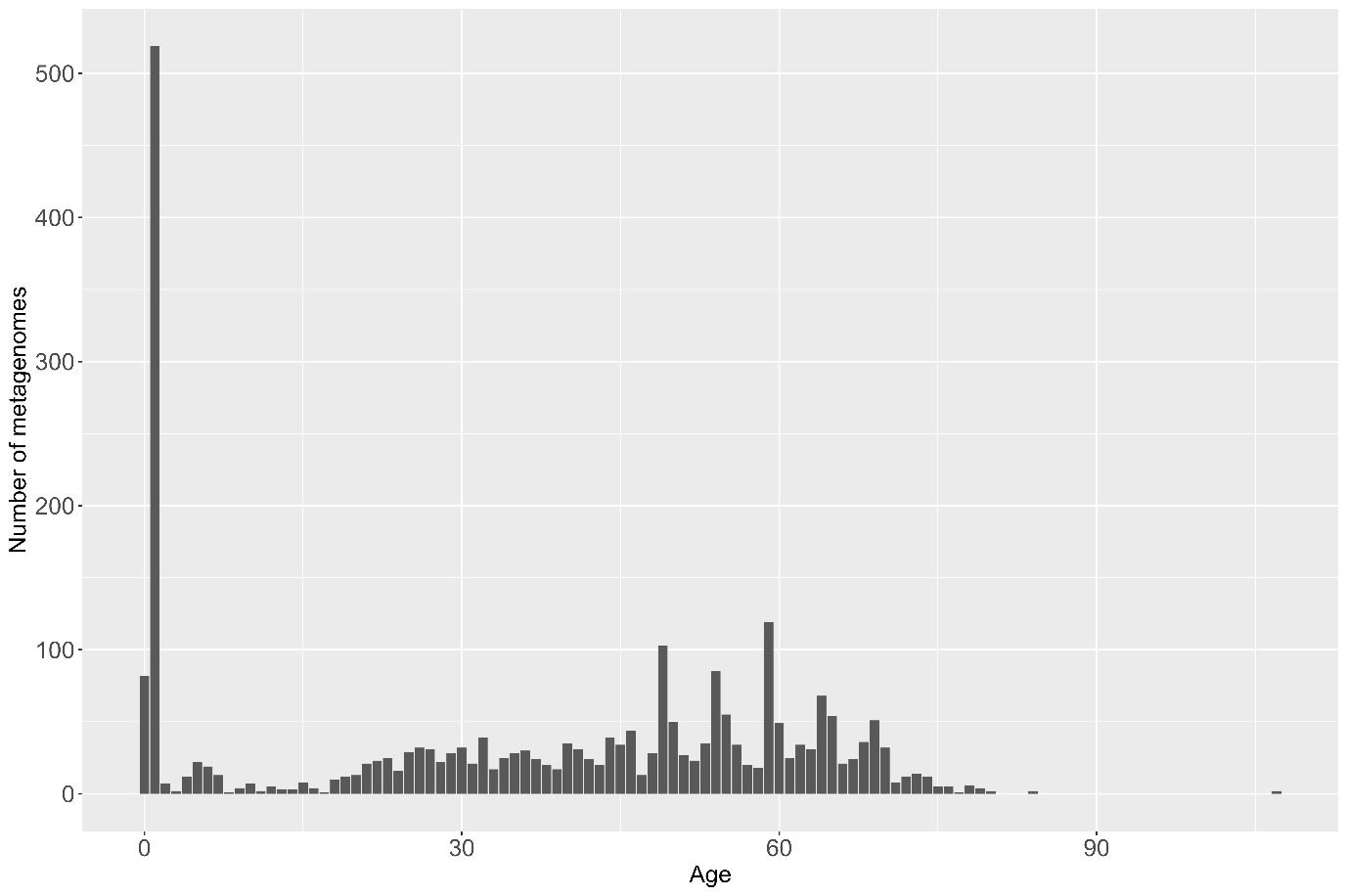
**

**Figure S6: Distribution of metagenomes:** The figure shows the number of metagenomes per age category.
